# Coping With the Experiences of Intimate Partner Violence Among South African Women: Systematic Review and Meta-Synthesis

**DOI:** 10.3389/fpsyt.2021.655130

**Published:** 2021-05-26

**Authors:** Yalda Sere, Nicolette V. Roman, Robert A. C. Ruiter

**Affiliations:** ^1^Department of Work and Social Psychology, Faculty of Psychology and Neuroscience, Maastricht University, Maastricht, Netherlands; ^2^Centre for Interdisciplinary Studies of Children, Families and Society, Faculty of Community and Health Sciences, University of the Western Cape, Bellville, South Africa

**Keywords:** intimate partner violence, coping, coping responses, mental health, violence, women, systematic review, South Africa

## Abstract

**Background:** Intimate partner violence (IPV) continues to be a serious problem worldwide. South Africa has a high prevalence of women experiencing IPV. Although much research reports on the prevalence rates, risk factors, and consequences of IPV, fewer studies report on how women deal with the experiences of IPV.

**Objective:** This systematic review of the empirical literature aimed to identify and synthesize the best available evidence on women's experiences of coping with IPV in South Africa.

**Methods:** A four-level search and retrieval strategy using PRISMA and JBI guidelines was conducted, which included critical appraisal, study selection, data extraction, and data synthesis. Ten studies met the eligibility criteria and were included in the review. They were assessed to meet a set threshold (7/10) based on the JBI Critical Appraisal Checklist for Qualitative Research. All studies were conducted between 2010 and 2020, conducted in South Africa, and used qualitative methodologies to accomplish the overall aim of investigating IPV experiences of women and their responses to it.

**Results:** The total number of women included in the studies was 159. The data extraction yielded 49 findings of which 47 were aggregated into 14 categories and three themes: (1) help- and support-seeking coping, (2) emotional regulation coping, and (3) problem avoidance and distraction coping. Help- and support-seeking coping refers to women's responses when they seek instrumental aid, advice, comfort, and/or understanding from others. Emotional regulation includes responses of women in which their emotions were expressed or regulated. Problem avoidance and distraction coping represent responses of women in which they take efforts to avoid thinking about the problem situation and rather reshift their focus.

**Conclusion:** Overall, this review found that a variety of coping responses are used by South African women experiencing IPV. The findings point to the need for understanding IPV and responses to it within a broader social context rather than just at the personal level. Approaching IPV at many levels may lead to a change in societal norms, better access to and delivery of services to IPV survivors, more functional family affairs, and personal well-being and improved quality of life.

## Introduction

### Intimate Partner Violence

Violence against women is historical, threatens the lives of women, and violates women's human rights. It cuts across nations, cultures, religion, and class and continues to this day. Although the majority of countries in the world have made violence against women a criminal act with societies at large condemning it, it continues to be a critical global problem. Most of this violence is intimate partner violence (IPV), defined as the experience of sexual, physical, or psychological harm by a current or former partner (1). Worldwide, approximately one-third (30%) of women who have been in a relationship state that they experienced some form of physical and/or sexual violence by their intimate partner in the course of their life (1). The consequences of IPV are negative, transcending health, social, and economic outcomes and include adverse well-being, employment, intergenerational impacts, and intrahousehold relations of women (2). The complex and multifaceted health consequences of IPV include physical, mental, sexual, and reproductive health issues, which, in turn, have implications for women's morbidity as well as mortality (1). At least one in seven homicides worldwide and more than a third of female homicides are committed by an intimate partner (3). One may differentiate between direct and indirect pathways through which IPV affects women's health. The former broadly includes injury, chronic pain, hypertension, sexually transmitted diseases, miscarriages, premature birth, and death (2). The latter includes psychological and physical stress, anxiety, trauma, reduced social functioning, and substance abuse (2). A multi-country study conducted by WHO (4) reported that emotional distress, suicidal thoughts, and attempted suicide were significantly higher among women who had experienced IPV compared with those who had not. Numbers of fatalities as a result of IPV are also very large. Moreover, IPV not only affects women, but all members of the family, which highlights the notion of treating violence in the family as a holistic phenomenon (5). UNICEF (6) estimates that worldwide 275 million children are exposed to domestic violence by either witnessing IPV and/or experiencing violence themselves.

South Africa is one of the highest-ranking countries in IPV prevalence with studies recording rates from 20% to 50% in which women report having experienced IPV at some point in their lives (7–9). IPV constitutes the second highest burden of disease in South Africa after HIV/AIDS (10). Accordingly, South Africa has among the highest reported femicide rates in the world with a femicide rate four times higher than the global rate (10, 11). Consequences of IPV are profound and far-reaching for South African women, including health, social, and economic effects. Research has shown that woman who experience IPV have increased risks for suffering adverse health outcomes (12), are more likely to attempt suicide (13), and are more likely to become infected by HIV than their counterparts in nonviolent relationships (14). Even though South Africa has recognized IPV as a punishable criminal offense since 1998 (15), it nonetheless remains among the highest ranking countries internationally in IPV prevalence as well as femicide (11).

### Coping With Intimate Partner Violence

Together with the importance of learning about IPV prevalence, consequences, and risk factors lies the significance of understanding how women respond to the violence they experience. Women may experience the same kind of violence, but the consequences in terms of mental and physical well-being may be different. One way to argue for such differences may be the way individuals cope with the incident(s), i.e., their coping strategies/mechanisms. Coping mechanisms have the potential to alter the impact of IPV on survivor's well-being (16). The strategies adopted by the survivor can either maintain well-being and, hence, mitigate the impact of IPV or expose her to greater degrees of risk. Examining coping strategies, therefore, is essential.

One of the most prominent conceptualizations of coping is presented by Lazarus and Folkman (17). They describe coping as thoughts and behaviors that people use to manage the internal and external demands of situations that are appraised as stressful (18). Further, a distinction is made between emotion- and problem-focused coping. Whereas, the former refers to regulating the distress connected to the particular problem and, thus, perceiving the situation as unchangeable, the latter refers to using strategies that manage the particular problem and, thus, perceiving the situation amenable to change (19). Another division frequently stated in the coping literature is the one between coping styles with an adaptive/healthy nature and those of a maladaptive/unhealthy nature (19). However, there is great difference in study findings when it comes to categorizing coping behaviors as either adaptive or maladaptive. This mainly depends on the type of stress or problem studied, the intensity and frequency of the problem, the study population, and cultural factors.

Globally, many researchers have addressed the coping responses of women experiencing IPV. Findings of these studies suggest that women use many coping strategies to manage substantial stress (20), escape reality (21), leave the violence in their lives (20), and establish safety for themselves (22). To identify coping responses that are mostly and rarely used, a systematic review was conducted by Rizo, Givens, and Lombardi (20) that examined 48 papers of studies conducted among female U.S. citizens. The authors found that the most common forms of coping included religious or spiritual coping, resisting the abuser, wishful thinking, trying to become more independent, maintaining relationships with others, and talking to others as well as leaving the abuser. Among the least commonly used coping responses were substance abuse, self-criticism, legal services, and seeking formal support (police, medical personnel, or a counselor). The participants in this study also rated the more frequently used coping responses as more helpful than the less frequently used coping responses. Contrary to the helpfulness ratings found in the described review, research and experts agree that seeking help from formal as well as informal sources constitutes an adaptive coping strategy (19, 23, 24). Additionally, hope, spirituality, and humor were found to be adaptive coping responses (24, 25). In contrast, substance abuse is considered a maladaptive coping response and may result in poorer health outcomes for the person (19, 24). Other maladaptive coping responses include mental disengagement, denial, and avoidance (19, 24, 25).

It is furthermore noteworthy to mention that factors such as age, ethnicity, employment, geographical location, and culture should not be disregarded in the discussion about coping. They all may play a role in the resources available to a woman and the coping responses she uses. Indeed, research regarding the socioeconomic status (SES) of IPV survivors has shown, for example, that IPV and poverty create parallel effects and constrain coping mechanisms (26). Both poverty and IPV elicit stress, powerlessness, and social isolation, which may evoke posttraumatic stress disorder (PTSD) and depression as well as other emotional problems. In turn, these factors inhibit the person to seek help. Another example regarding one's geographical location and culture is reported by Horn (27), who indicates that access to services in rural areas is limited and that access to services may be more difficult due to governmental policies and societal norms. Thus, a woman's coping response to the violence she experiences depends also on the options available to her. Differences in culture with respect to environmental demands, social structure, resources, and cultural norms may also influence coping strategies. A review conducted by See and Essau (28) found that there is a stronger tendency for collectivistic countries toward emotion-focused coping in comparison to individualistic (Western) countries.

### The Current Study

The aim of the present study is to provide a comprehensive systematic review and synthesis of the available literature on women's experiences of coping with IPV in South Africa. We focus on both adaptive and maladaptive coping in this review. The feminist theory and the social-ecological framework serve as theoretical frameworks to understand IPV and its causes, risk factors, and coping responses. Whereas, a growing body of research has examined the prevalence rates that different forms of IPV have and risk factors associated with them, rather little research has examined the strategies survivors have used to cope with the violence, especially in the South African context. Much of our understanding of coping is based mainly on Western cultures (28). However, as South Africa has among the highest rates of IPV globally, it is essential to have a closer look at the coping strategies in this country as well. Thus, this review aims to establish filtered evidence comprising high-quality qualitative studies that explore the coping experiences of women facing IPV in South Africa. The focus of the current study on only qualitative studies lies in the exploratory nature of qualitative research. An initial search of studies revealed that there has been no other systematic review conducted on this topic in the South African context. Therefore, there is a lack of filtered studies, both quantitative and qualitative, whose methodologies have not been rigorously and systematically evaluated along specified criteria. Together with insufficient filtered literature as well as considerable ambiguity about the topic, it is difficult to understand and sufficiently address how women respond to violence in South Africa. Investigating what is known so far qualitatively about coping with IPV may be useful for informing future quantitative research aimed at assessing effective ways of how women deal with the aftermath of violence. Further, a systematic analysis of the qualitative evidence may be important for the careful and detailed development of interventions and programs at the level of individual beliefs and influences from social and physical environments in which South African women live.

## Materials and Methods

The study utilizes a systematic review methodology. Systematic reviews are defined by collecting all possible studies related to a given topic and design and reviewing and analyzing their results (29). They aim to provide a comprehensive and unbiased synthesis of “all” evidence about a particular question in a standardized, systematic way (30). The systematic review is carried out by taking into account the recommendations by PRISMA and guidelines of the JBI Manual of Systematic Reviews of qualitative evidence.

### Search Strategy

A search strategy was designed using the population, exposure, and outcome (PEO) framework. The population of interest were South African women experiencing IPV. Hence, the exposure constitutes experiencing IPV. Last, the outcome refers to coping with IPV. The PEO framework, thus, yielded the following research question: What are the coping strategies used by South African Women experiencing IPV?

The following electronic databases were searched to identify potential studies: Web of Science (WoS), PubMed, EBSCOhost, and Google Scholar. Although the first three databases were searched on the same date (May 18, 2020), the fourth was searched 4 days later (May 22, 2020). The databases yielded 735 hits in total of which 334 were generated by WoS, 247 by PubMed, 13 by EBSCOhost, and 141 by Google Scholar using the following key terms and Boolean strings: intimate partner violence OR domestic violence OR partner abuse AND coping OR coping strategy OR responses OR coping mechanism OR experience AND South Africa. The specific search strings matching the database are displayed in Appendix 1.

### Inclusion and Exclusion Criteria

To identify and select the studies most relevant to the present review, inclusion and exclusion criteria were established. Inclusion criteria were (i) the paper focused on qualitative data, including but not restrictive to designs, such as phenomenology, feminist research, and discourse analysis; (ii) the paper explicitly included or discussed women above the age of 18 who had personally experienced IPV; and (iii) papers were conducted between 2010 and 2020. Exclusion criteria were (i) the paper was in a non-English language, (ii) the study was not conducted in South Africa, and (iii) papers were not conducted in the designated time frame.

### Study Selection

**Figure 1:**
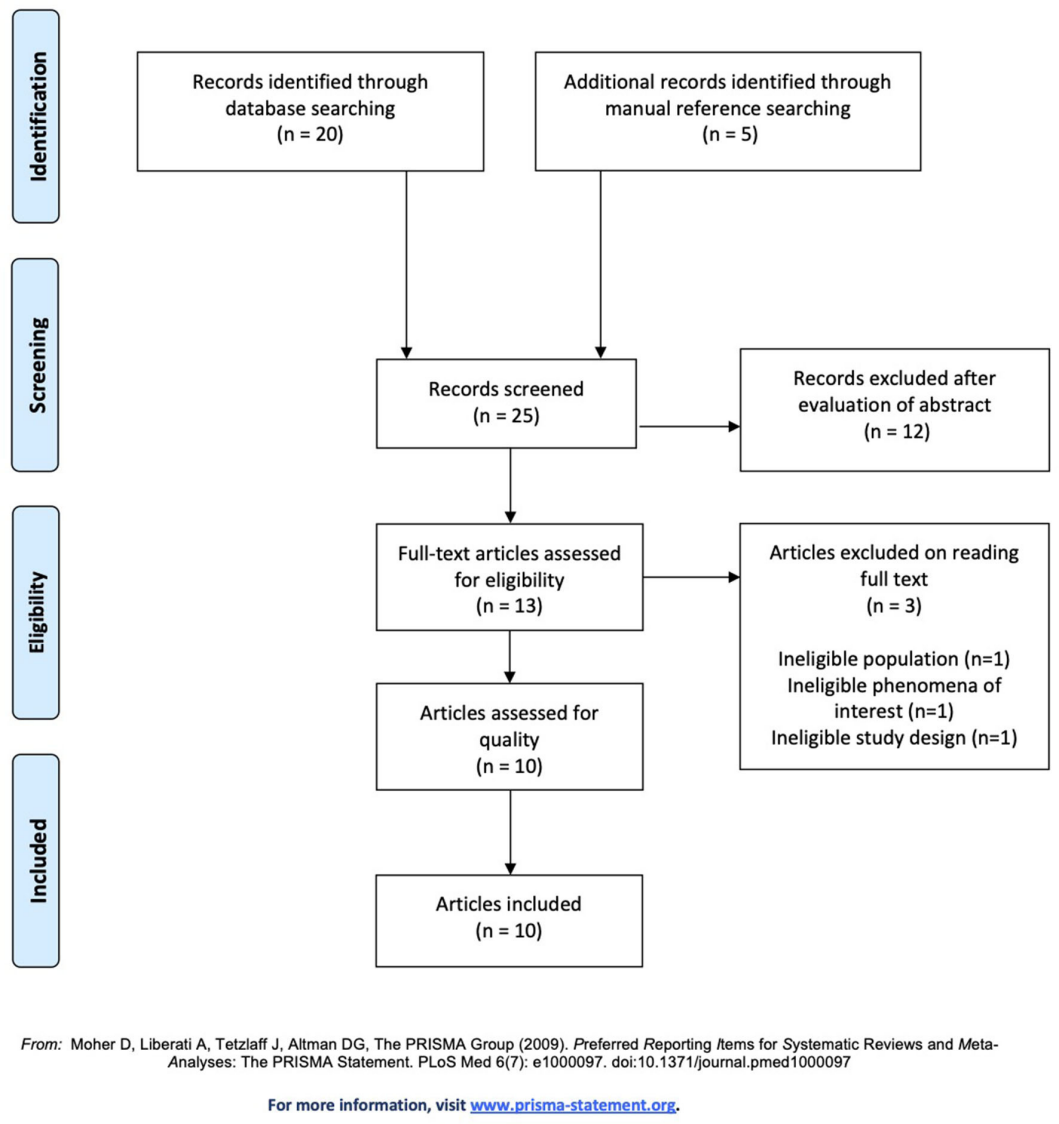
PRISMA flow diagram (31).

Out of the 735 hits yielded by the databases, potential titles were screened in each database, resulting in 20 studies (WoS = 5, PubMed = 7, EBSCOhost = 2, and Google Scholar = 6). Additional studies were identified from the reference list of all articles considered. The title search of the reference lists yielded five potential studies. Abstracts of 25 studies were screened for further inclusion by means of meeting the abovementioned inclusion as well as exclusion criteria of the study. This led to 12 articles being excluded after evaluation of the abstract. The remaining 13 records were retrieved for a full text review also with regards to the inclusion and exclusion criteria. Three articles were excluded in this step due to one article having the wrong study design, another having an ineligible population, and one for not focusing on the phenomena of interest. This process led to a final selection of 10 included studies to be assessed for methodological quality. The PRISMA flow chart in Figure 1 shows the selection process of the included studies.

### Quality Assessment

The methodological quality of the studies was assessed using the JBI Critical Appraisal Checklist for Qualitative Research (32), and ratings were conducted by two researchers for all articles (double coding). Disagreements in ratings were discussed until consensus was reached. The JBI Critical Appraisal Checklist offered a framework for assessing the quality of the potential studies by addressing different aspects of the research. Ten questions regarding ethics, possible biases, the integrity of the methodology and congruity between methods, results, and conclusion were rated. The researchers set a threshold score (7/10) to determine whether a study should be further included in the review or not. Out of the 10 methodological assessment questions, seven had to be met with a “yes” (as opposed to “no” or “unclear”) to be included in the final level. Table 1 shows the critical appraisal of the included studies. Decisions about the cutoff score for exclusion were made in advance and agreed upon as suggested by the JBI manual. All studies that met the designated threshold score were next subjected to the data extraction process using the standardized JBI Qualitative Assessment and Review Instrument (JBI-QARI) (32). The QARI tool provides a structured data extraction sheet to promote the extraction of similar data across all of the included studies as well as another extraction sheet for extracting findings and their credibility (32). The first extraction tool includes specific details about the populations, context, culture, geographical location, study methods, and phenomena of interest relevant to the review question. Table 2 shows this extraction for all the included studies. The second extraction tool in regard to findings and their credibility is explained below.

**Table 1:** Critical appraisal of included studies.

**Included studies**	**Q1**	**Q2**	**Q3**	**Q4**	**Q5**	**Q6**	**Q7**	**Q8**	**Q9**	**Q10**	**Sum**
Slabbert (33)	Y	Y	Y	Y	Y	N	N	Y	Y	Y	8/10
Maselesel (34)	Y	Y	Y	Y	Y	N	N	Y	Y	Y	8/10
Van der Merwe and Swartz (35)	U	Y	Y	Y	Y	N	N	Y	Y	Y	7/10
Rasool (36)	Y	Y	Y	Y	Y	U	U	Y	N	Y	7/10
Mkhonto et al. (37)	U	Y	Y	Y	Y	Y	N	U	Y	Y	7/10
Boonzaier et al. (38)	Y	Y	Y	Y	Y	N	N	Y	N	Y	7/10
Baholo et al. (39)	U	Y	Y	Y	Y	N	Y	Y	Y	Y	8/10
Rasool (40)	Y	Y	Y	Y	Y	Y	N	Y	N	Y	8/10
Dekel and Andipatin (41)	Y	Y	Y	Y	Y	N	N	Y	Y	Y	8/10
Chikwira (42)	Y	Y	Y	Y	Y	N	N	Y	Y	Y	8/10

*N, No; U, Unclear, Y, Yes*.

*Criteria for the critical appraisal of qualitative evidence:*

*Q1 = Is there congruity between the stated philosophical perspective and the research methodology?*

*Q2 = Is there congruity between the research methodology and the research question or objectives?*

*Q3 = Is there congruity between the research methodology and the methods used to collect data?*

*Q4 = Is there congruity between the research methodology and the representation and analysis of data?*

*Q5 = Is there congruity between the research methodology and the interpretation of results?*

*Q6 = Is there a statement locating the researcher culturally or theoretically?*

*Q7 = Is the influence of the researcher on the research, and vice-versa, addressed?*

*Q8 = Are participants, and their voices, adequately represented?*

*Q9 = Is the research ethical according to current criteria or, for recent studies, and is there evidence of ethical approval by an appropriate body?*

*Q10 = Do the conclusions drawn in the research report flow from the analysis, or interpretation, of the data?*

**Table 2:** Data extraction.

**Reference**	**Methodology**	**Method**	**Setting/country**	**Participants**	**Phenomena of interest**	**Data analysis**	**Phenomena of interest**
Slabbert (33)	Phenomenology	Semi-structured in-depth interviews	Four interviews were conducted in “the Wimpy,” five in the researcher's car, nine at the clinic, two at a psychologist's office, Cape Town, SA	20 adult women	Coping mechanisms and resources of abused women	Thematic analysis	Five coping strategies(strengths) found: 1) Children 2) Religion 3) Hope 4) Survivor 5) Alcohol Three coping resources: 1) Significant others (family, friends, and neighbors) 2) Church (pastor, church members) 3) Professional help (social worker, clinic sister, and psychologist)
Maselesel (34)	Phenomenological, qualitative, descriptive	Phenomenological Interviews	Interviews were held in a private place	18 adult women	Women's experiences of coping with domestic violence	Thematic analysis	There are six stages ofcoping: 1) Self-blame/denial 2) Apathy 3) Uncertainty 4) Acceptance 5) Anger and retaliation 6) Self-discovery
Van der Merwe and Swartz (35)	Qualitative	Semi-structured interview	A shelter for female survivors of IPV in Maneberg, Cape Town, SA	4 adult women	Experiences of IPV	Narrative analysis	Coping: -Religion -Support from family -Counseling -Substance abuse
Rasool (36)	Feminist research	In-depth interviews	Conducted in abused women's shelters in Cape Town and Johannesburg, SA	17 adult women	Love as a central factor in the experience of IPV	Life-history analysis	Love is a powerful reason that keeps women trapped in abusive relationships. Abused women stay because their partners are not abusive all of the time and in some instances are able to display caring and loving behaviors
Mkhonto et al. (37)	Qualitative	In-depth semi-structured interviews	Interviews done in an admission ward at the medico-legal/crisis center of a public hospital in Tshwane, SA	10 adult women	Experiences of women on IPV	Content analysis	Coping mechanisms: 1) Internal motivation 2) Spirituality 3) Children
Boonzaier et al. (38)	Discourse analysis	Semi-structured and unstructured face to face interviews	Urban (29) and semi-rural (15) areas of Cape Town, SA	44 adult women	Women's strategies on telling about IPV experience	Thematic narrative analysis	Women fight back; Women have their internalized roles (good wives, mothers, homemakers) they want to keep
Baholo et al. (39)	Qualitative	In-depth face to face interviews	All interviews took place in an abused women's shelter in a private room Gauteng Province, SA	11 adult women, 10 of African descent, and 1 of Indian descent	Women's experiences facing IPV	Thematic analysis	A phase of change (progression of violence, realization the partner would not change, the effect of abuse on the children and the women's own feelings due to abuse) and the process of leaving the abusive relationship (a supportive environment, access to shelter, opportunity to leave)
Rasool (40)	Feminist research	In-depth interviews	Interviews were conducted in domestic violence shelters in JHB and Cape Town, SA	17 adult women	Women's experiences of help-seeking	Content analysis	Influences of powerful social discourses on the best interest of the child
Dekel and Andipatin (41)	Feminist poststructuralism and discourse analysis	Open-ended interviews	Interviews were held in private rooms of an abused women's shelter in Cape Town, SA	7 adult women	Experiences of women survivors of IPV	Discourse analysis	Women drew on discourses of femininity, romantic love, environmental support, dissociation, normalization, self-blame, and religion to endure
Chikwira (42)	Intersectional analysis	Semi-structured face to face interviews	Interviews were conducted in a room in an abused women's shelter in Cape Town, SA	11 adult women	Life histories of IPV survivors	Thematic narrative analysis	Women cope through minimization and normalization

According to the abovementioned methodological assessment, a total of 10 studies were recognized as appropriate for the purpose of the current study and were included in the final review to address the objectives and research question of the present study. For the data extraction, each of the 10 identified studies were coded in a preformulated data extraction sheet according to the following characteristics: first author name, year conducted, methodology, methods, setting, phenomena of interest, data analysis, and the authors' conclusion. Next, findings of each included study were described in a consistent manner to synthesize and interpret them at a later stage. A level of “credibility” was given to each finding based on the amount of support presented by each illustration of the finding. As described by the standardized JBI qualitative extraction tool the below presented levels were given. A full list of findings along with illustrations and levels of credibility are presented in Appendix 2.

- Unequivocal (U): the findings' accompanied illustration is not open for challenge.
- Credible (C): the findings' accompanied illustration is open for challenge.
- Unsupported (US): the finding is not supported by data.

### The ConQual Approach

The ConQual approach, also called “summary of findings” is a systematic method to consider what increases or decreases confidence in the results of the qualitative studies (43). This depends on the type of the research, the dependability of the study, and the credibility of findings. The analysis of these criteria results in a ConQual score (high, moderate, low, and very low). The analysis starts off by pre-ranking the papers from (1) high, (2) moderate, or (3) low to (4) very low. In this pre-ranking, qualitative studies are considered high, and text and opinion papers are ranked low (32). From this starting point, each paper is then graded for dependability and next for credibility. Depending on the specific criteria for both dependability and credibility, the initial ranking either stays the same or moves down to one or more levels.

Dependability is measured by asking questions regarding the appropriateness of the conduct of the research. Questions two, three, four, six, and seven of the abovementioned JBI Critical Appraisal Checklist are asked (see Table 1). If four to five “yes” responses are given, the initial ranking of the paper remains the same. Two to three “yes” responses lead to moving down one level in the ranking. Zero to one “yes” responses lead to moving down two levels in the ranking. Credibility is measured by cross-checking how many findings of which level of credibility (unsupported, credible, and unequivocal) were included in the categories associated with the synthesized finding. If a synthesized finding consists of only unequivocal findings, the ranking (yielded in the dependability analysis) remains unchanged. However, if a synthesized finding consists of a mix of unequivocal and credible findings, only credible, or a mix of credible and not supported findings or, last, of only not supported findings, the ranking (yielded in the dependability analysis) is downgraded accordingly (−1, −2, −3, and −4).

All included studies were of qualitative design and, therefore, received an initial ranking of “high.” For all three synthesized findings, the majority of the included studies received two to three “yes” responses on the ConQual identified criteria for dependability; therefore, the ranking moved down to one and yielding a “moderate” level of confidence. Credibility levels of the second and third synthesized findings were downgraded one level due to a mix of unequivocal and credible ratings, and the first synthesized finding remained unchanged due to only unequivocal ratings. The so-called summary of findings can be seen in Table 3.

**Table 3:** ConQual summary of findings.

**Synthesized finding**	**Type of research**	**Dependability**	**Credibility**	**ConQual score**	**Comments**
**Help- and support-seeking coping**Women's experiences of IPV led them to seek instrumental aid, advice, comfort, and/or understanding from others. They recognized these people as significantly supportive.	Qualitative- High	Downgraded one (−1)^*^	No change^**^	Moderate	^*^The majority of studies (three out of five) scored three out of five for the questions relating to appropriateness of the conduct of the research; therefore, the dependability score has downgraded one leading to a ranking of moderate. ^**^No change due to only unequivocal (U) findings leading to a final ranking of moderate. U = 10
**Emotional regulation coping**Women experience a number of emotions as they go through IPV. They take effort keep them under control, let them out, or reconstruct their emotional understanding.		Downgraded one (−1)^*^	Downgraded one (−1)^**^	Low	^*^The majority of studies (six out of seven) scored three out of five for the questions relating to appropriateness of the conduct of the research; therefore, the dependability score has downgraded one leading to a ranking of moderate. ^**^Downgrade one level due to mix of unequivocal (U) and credible (C) findings leading to a final ranking of low. U = 9, C = 7
**Problem avoidance and distraction coping**Women exposed to IPV take effort to distract themselves, avoid and/or shift their focus on the abusive behavior of their partner.		Downgraded one (−1)^*^	Downgraded one (−1)^**^	Low	^*^The majority of studies (five out of eight) scored three out of five for the questions relating to appropriateness of the conduct of the research; therefore, the dependability score has downgraded one leading to a ranking of moderate ^**^Downgrade one level due to mix of unequivocal (U) and credible (C) findings leading to a final ranking of low. U = 6, C = 10

### Data Analysis

The research findings were pooled by means of the meta-aggregation approach described by the JBI Manual of Systematic Reviews of Qualitative Evidence (32). It involves a three-step process of data synthesis described below:

- Findings extraction from all included papers with an accompanying illustration and assigned credibility level for each finding.
- Categorization of findings based on similarity in meaning and concepts.
- Development of a comprehensive set of aggregated findings (of at least two categories) that could be used as a basis for evidence-based practice.

The synthesized findings were evaluated with the abovementioned ConQual approach as presented in JBI's Manual to establish a level of confidence in each synthesized finding (Table 3).

### Meta-Synthesis

For each included study, findings were extracted using the extraction sheet of the abovementioned JBI-QARI. The findings were extracted with an illustration from the original data and assigned a level of credibility. A finding is defined as a literal extract of the authors analytic interpretation, which is supplemented by either a participant's voice or other data (32). Accordingly, an unequivocal finding is accompanied by an illustration that demonstrates the authors interpretation beyond doubt. A credible finding is also underpinned with an illustration; however, the two lack clear association, and the interpretation of the author can be challenged. An unsupported finding is not demonstrated with any illustration (32). In this review, only unequivocal and credible findings have been included in the synthesis of data as recommended by the JBI manual.

Hence, each finding of the studies was assigned a credibility level and supported by at least one illustration from the study. For example, finding 7 was *Alcohol as a Coping Mechanism*. This was supported by two illustrations from the study as follows: “I take a doppie. That's how I cope.” (participant X) or “You take a doppie and dance just to try to get through life and to forget the pain.” (participant Y) (33). Both illustrations support the authors' finding and present a minimal risk of misinterpretation; thus, they were considered to be unequivocal. In total, 49 findings were found, and the same process as described was followed. A small majority (25) of the findings (51%) were unequivocal (U), 22 (45%) credible (C), and two (4%) unsupported (US). As JBI does not recommend the inclusion of unsupported findings, the two unsupported findings were not included in the next stages of the meta-synthesis. A full list of findings accompanied by illustrations can be found in Appendix 2. The 49 findings were repeatedly read and reread to compare and identify similarities between them. Those found to be similar were aggregated into 14 categories, namely (1) children, (2) religion, (3) harmful substance use, (4) informal support, (5) formal support, (6) self-blame, (7) love, (8) hope, (9) anger/fight, (10) dissociation, (11) internalizing harmful gender roles, (12) normalization and minimization, (13) acceptance, and (14) no category. A full list of findings and categories is presented in Appendix 3. The categories were further examined to identify if they could be synthesized according to similarity in meaning and grouped into themes. This process was done using the thematic analysis defined by Braun and Clarke, which covers six phases: familiarizing with the data, generating initial codes, searching for themes, reviewing themes, defining and naming themes, and writing the report (44). In this review, a synthesized finding or theme includes at least three findings that are similar in meaning. The synthesis of the findings into categories yielded the following three themes: (1) help- and support-seeking coping, (2) emotional regulation coping, and (3) problem avoidance and distraction coping.

## Results

### Study Characteristics

All 10 studies included in the present review were conducted between 2010 and 2020, conducted in South Africa, and used a qualitative design. Six studies were conducted in the Western Cape Province (Cape Town), three studies in the Gauteng Province (Johannesburg and Tshwane), and one study in the North West Province (Mafikeng). The total number of participants included in the studies was 159. Appendix 4 gives an overview of the participants' characteristics. It is noteworthy to mention that the race categories describing the women of the studies originated and developed under Apartheid. Although they are not unproblematic, they are still frequently used today in research. The following characteristics were found for the 10 included qualitative papers. The methods included in this review were phenomenology (two), feminist research (three), discourse analysis (two), and unspecified qualitative (three). All studies focused on solely women's accounts of their experience with IPV, investigating their understanding and responses to it. Five studies specifically investigated coping responses of South African women to deal with IPV. The other five studies aimed to explore the experiences of IPV in general. Among several identified experiences, they also found coping experiences of women dealing with IPV. Interview settings were a private room in abused women's shelter (seven studies), participants' homes (one study), a private room in a medico-legal/crisis center of a public hospital (one study), or unspecified (one study). Data collection methods used were mainly semistructured or in-depth, face-to-face interviews. Data analysis methods were coherent with the qualitative methodology used in each study.

### Themes

#### Help- and Support-Seeking Coping

Three categories comprising 10 findings were integrated into the first theme, which can be seen in Table 4, help- and support-seeking coping. The first category, *informal support*, refers to the people to whom women reach out to find support for their difficult situation. These include family members, friends, and neighbors. Participants found these people to be very supportive as shown with this statement: “My mother is my biggest support. If it was not for her, I would not cope” (33). The second category, *formal support*, refers to professional people to whom women reach out for help, including police officers and counselors. Some women described how the counselor, for example, made them feel stronger (“the counselor has made me stronger” or “my psychologist tells me regularly what a strong woman I am”) (33, 35). The last category, *religion*, refers to people in the church, including the pastor as well as church members and also God. Many participants stated how God had helped them cope by, for example, stating “[the Lord] he carries me through this difficulty” (33). All three categories represent a type of response in which women seek instrumental aid, advice, comfort, and/or understanding from others.

**Table 4:** Theme 1: Help- and support-seeking coping.

**Findings**	**Categories**	**Theme**
Participants found their family, friends, and neighbors to be very supportive [U]	Informal Support	*Help- and support- seeking coping*
They turned to family and friends during those times when they were desperate for support [U]		
Drawing on police services for help and assistance [U]	Formal Support	
Use of some form of professional [U]		
Coping mechanism included counseling [U]		
Religion is one way of helping them cope with their difficult situations [U]	Religion	
The church was noted as providing support and a way of coping (pastor and members of church) [U]		
Coping mechanism included religion [U]		
Resilient due to their spirituality [U]		
Positioning with religious discourses [U]		

*U, Unequivocal*.

#### Emotional Regulation Coping

The meta-synthesis into emotional regulation coping resulted from five categories, comprising 16 findings that can be seen in Table 5. The first category was *love*. Several women in the studies described how, on the one hand, loving their partner had helped them deal with the abuse, and on the other hand, loving behaviors from their abusers helped them get through. Love seemed to be a powerful concept that enforced women to survive their relationship as one woman clearly stated: “He tells me he loves me (.) he was my reason for living (.).” (36). The second category, *self-blame*, refers to women transferring the guilt of the abuse onto themselves. They believe that they are to blame for their partner's violence, either for doing something wrong (“I shouldn't have talked to him”) or for choosing the wrong partner (“I always went for men that had the tendency of abusiveness”) (34, 42). There were also some women who identified *hope* as a coping mechanism, which formed the third category. Hopes about the abusive relationship becoming “better” as well as hopes about the partner changing were communicated by the women: “I hope that everything will come right. It is hope that lets me go on.” (33). The next category, *anger/fight*, refers to women identifying feelings of anger and fighting back as coping with their abuse. Several women expressed how they fought back or how they imagined retaliating their partner (“I wish I could kill him before he kills me”) (34). The last category, *acceptance*, refers to those women who communicated enduring the relationship due to their accepting position of the situation (“I made a commitment, I will not go away”) (34).

**Table 5:** Theme 2: Emotional regulation coping.

**Findings**	**Categories**	**Theme**
Love women feel for the abuser [U]	Love	*Emotional regulation coping*
Abuser displays caring and loving behavior [U]		
Maintain the romantic fairy-tale ideal (love) [C]		
Love influences a woman's commitment to remain in a relationship, albeit an abusive one [U]		
The victim blames herself that she is responsible for the abuse [C]	Self-blame	
She deserves to be treated in that way [C]		
The women tended to blame themselves for their partner's violence, and accepted their partner's blaming of them [U]		
Place blame of experiencing IPV on themselves (self-blame) [C]		
They had hope and identified that as a coping mechanism [U]	Hope	
Hope that relationship will return to a better time [C]		
Participants believed that their partners could change [U]		
Women retaliates and is prepared to fight [U]	Anger/Fight	
Fighting back [U]		
Anger at the abuse [U]		
Victim accepts there is nothing she can do about the abuse [C]	Acceptance	
Enduring the relationship [C]		

*U, Unequivocal; C, Credible*.

#### Problem Avoidance and Distraction Coping

Six categories comprising 16 findings were integrated into the last theme of problem avoidance and distraction coping and can be seen in Table 6. The first category, *harmful substance use*, refers to women coping with the help of alcohol. Women describe how the use of alcohol made them forget the pain as one participant stated: “You take a doppie and dance just to try to get through life and to forget the pain” (33). The second category, *dissociation*, refers to how women project the partners responsibility of the abuse onto something else, which helped the women to see the abuse and the abuser in a different light. *Internalizing harmful gender roles* formed the third category and refers to women ascribing themselves to different roles. Most women referred to their role as a good wife, mother, and homemaker as the following statement of the participant states: “He needed me to clean him up, take care of him, of his children (…)” (38). Also, societal expectations of how a family should be came up as one woman expressed her concerns for the upbringing of her children in saying: “I didn't want my children growing up without a father” (40). The fourth category, *normalization and minimization*, refers to women downplaying the abuse or evaluating it as normal (“Maybe it should be like that” or “The hitting is not so bad”) (41, 42). *Children* formed the fifth category. Here women expressed how their children helped them get through as their love for them inspired them (“It's just my children. I love them very much. They are the reason I am going on”) (33). The last category, *motivation*, refers to women motivating themselves in some aspect to cope with their abusive relationship. For example, participant X stated, “I am special (.) I have managed to reach grade 12, there are many chances that I can get so that I become better,” or as another woman said: “I will get to the top (…) I am a fighter (.)” (33, 37). All categories represent responses of women in which they take efforts to avoid thinking about the problem situation and rather reshift their focus mostly by using some sort of distraction as well as reorganizing the way they look at the problem situation.

**Table 6:** Theme 3: Problem avoidance coping and distraction coping.

**Findings**	**Categories**	**Theme**
Participants indicated that alcohol helped them to forget and to cope [U]	Harmful substance use	*Problem avoidance coping and distraction coping*
She denies that her partner is responsible for his actions [C]	Dissociation	
To cope with the abuser, she finds excuses for her abuse [C]		
Many women dissociate the abuse from the “real man” and attributing it rather to factors that he does not have control over [C]		
Emphasized femininity or good womanhood (role as good wives, mothers, and homemakers) [U]	Internalizing harmful gender roles	
Fulfilling the role of mother [C]		
Internalization of dominant prescriptions of femininity [C]		
Preserving the two-parent family form [C]		
IPV is normalized [U]	Normalization and minimization	
Normalization of IPV [U]		
Minimization of IPV [U]		
Children helped these women to cope [U]	Children	
Their children were their goal in life and helped them go on [C]		
Children as a source of inspiration [C]		
Some abused women are able to identify their own courage, wisdom, and resilience and are able to view themselves as capable human beings [C]	Motivation	
Resilient due to their inherent motivation [C]		

*U, Unequivocal; C, Credible*.

## Discussion

### Summary of Main Findings

The synthesized findings show that women who experience IPV use a wide variety of coping mechanisms to deal with the violence. Consistent with the prominent conceptualization of Lazarus and Folkmann (17), which reports that coping mechanisms may be problem- or emotion-focused, the current findings also yield problem- and emotion-focused coping strategies with the former referring to responses aimed at changing the problem and the latter to responses aimed at altering the feeling associated with the problem. However, there was a slight tendency toward emotion-focused coping, which aligns with previous research stating that collectivistic countries have stronger tendencies toward emotion-focused coping (28).

Many women in the present study responded to the violence they experienced by seeking help and support from others. Such responses have been linked to problem-focused coping strategies in previous research as they have the potential to alter the problem (19, 45). Previous research has also reported that informal networks were identified by survivors of IPV as very helpful (23). This is in line with the results of the present review in which women reported to find their family, friends, and neighbors as very supportive. This highlights the important role of community- and interpersonal-level impacts that should be considered in IPV research. It also supports the view that IPV should not be looked at as an individual problem but rather considered a community problem. Women in the present study also reported to have drawn on police services for safety and assistance as well as using counseling sessions by psychologists and social workers. These findings are consistent with other studies identifying formal support as a coping mechanism (19, 46). In addition, the present review found that many women reached out to religious support by consulting pastors and talking to members of the church. They also stated that “God” was a source for coping with abuse. Similarly, previous research has identified religion and spirituality as coping mechanisms of IPV survivors (47).

Together, these findings show that women are not passive survivors but rather active in that they adopt active strategies to ensure their safety and comfort through the help of others. Research often reports that women suffer in silence (48). However, the present findings show that the survivors are not silent, but instead active in sharing their experiences with others as well as seeking out help from others. Another important implication of the findings is to view IPV from many levels (interpersonal, community, and society) instead of perceiving it solely as a personal issue. Understanding IPV only at the personal level can be dangerous as it may contribute to an overall lack of the understanding of survivor's situations. It may also preserve the view that the responsibility of an abuse remains only on women, which, in turn, may perpetuate privatization of abuse. Generally, it is often assumed that the more a society openly addresses an issue at all levels, the more room there is for change. It is an open question if this is also the case in South Africa.

The current review further found that many coping responses of women experiencing IPV involved focusing, expressing, or shifting their emotions. For example, women identified love as a powerful survival strategy. They reported that the love they felt for their partner kept them going (reason to survive), which also influenced their commitment to stay in the abusive relationship. This is not uncommon as the concept of love has been linked to a woman's decision and process of leaving an abusive partner in previous research. In a study conducted by Copp et al. (49), they found that love was a significant factor that influenced an individual's decision to leave or stay in an abusive relationship. In addition, the present review found that many women blamed themselves for the abuse they were experiencing. Several women reported feeling responsible for their partners' violence. One woman even stated that she deserved to be treated in that way. The concept of self-blame has been identified as a frequent emotion-focused strategy of women experiencing IPV (19, 50). The research also notes that self-blame constitutes a maladaptive strategy as it can increase negative affectivity as well as lack of behavior change. For women survivors of IPV, this may mean not leaving the abuser. Also, self-blaming feelings may contribute to the help-seeking behavior of IPV survivors. Findings of a literature review found that, among others, feelings of self-blame too formed a barrier to seek help from formal as well as informal networks (51). Furthermore, similar to findings identifying hope as an emotion-focused coping strategy, this review also found hope to be a coping mechanism for several women (52). The beliefs included hope about a better relationship in the future as well as hope about a behavior change in their partner. This is an important finding because it shows that, like other emotion-focused coping responses, hope has the potential to shift the negative feelings of women and instead view the relationship in a more positive light. However, women in this study also expressed anger toward their abuser and wishes about fighting back as well as actual fighting back. This is an example of a response in which women actively let their emotion out and let their abuser know about their anger. Similarly, a recently conducted study found that a strategy of women dealing with IPV was the use of self-defense and fighting back, which they further identified as an effective strategy (53). However, although some studies report that fighting back was perceived as effective by IPV survivors, many other studies report that this strategy was identified as extremely ineffective (53). Independent of the effectiveness, the importance of the present finding is that it, once again, shows that women experiencing IPV can show agency and that they are able to defend themselves. It is noteworthy, however, that agency should not solely be measured by a women's capability to fight back as many other factors influence such a decision, and consequences of displaying such a type of agency may result in unimaginable consequences. Some women in this study also reported having accepted their “fate” and decided that there is nothing they can do about the abuse. This finding is consistent with other studies reporting that IPV survivors accepted what happened and what might happen to them (47, 54). This form of response has also been identified as an emotion-focused strategy as it reconstructs the way a person looks at the problem situation rather than addressing the problem itself (19).

The reported findings of the current review highlight that women use a range of emotional coping responses. They express their emotions, keep them under control, and/or reconstruct the way they emotionally understand the problem situation. However, many of the responses, as previous research also shows, are not necessarily effective as they can negatively impact decision making. Yet, it is important to acknowledge the presence of emotions in survivors' situations and recognize their influence on decision making. This is especially important for organizations providing services to survivors of IPV who rely on the decision-making processes of women.

Many coping responses of women in the present study were characterized by avoidance and distraction, which confirms previous research suggesting that women deal with the violence they experience by avoiding it or distracting themselves (47, 54, 55). A longitudinal study conducted in 2015 found that IPV survivors drank to cope with IPV (56). This finding is reflected in the current review as women identified the use of alcohol as a coping mechanism, which constitutes a maladaptive coping response (19). Moreover, women identified dissociation as a way of coping in that they projected their partners' responsibility for the abuse onto something else. This led to them reorganizing the way they see the abuse and the abuser. Hence, they dissociated the abuse from the “real man” and instead attributed the abuse to factors over which the abuser did not have any control. Women in the present study also ascribed different roles onto themselves. Those roles mostly emphasized femininity or womanhood in the form of being a good wife, mother, and housekeeper. The internalization of such prescriptions was weighted heavier by the women than the abuse they were facing. Also, societal expectations of how a family should look were evident as women expressed their concerns about raising a child without a father and being a single parent. This is in line with studies reporting that women feel a responsibility to maintain the marriage and family and shame associated with being divorced or being unmarried (57). Importantly, these findings suggest that societal norms may result in women enduring IPV silently due to the cultural pressures of preserving the status quo and sustaining a two-parent family form. It is important to note, however, that gender roles are foremost fundamentally a cause of IPV in that they constitute community and societal risk factors of IPV (58). These gender roles influence relationships between men and women. This influence is seen in that violence against women within a couple is being legitimized within the context of adherence to traditional gender norms and roles (59). Such legitimization from the women's perspective can be understood as a form of maladaptive coping (59). Research also suggests that norms facilitate the normalization of IPV (60), which was also evidenced in the present review. Several women stated that IPV was “normal” and just part of life. This finding can be seen as a direct mirror of societal norms accepting and tolerating IPV and considering it a normal, everyday occurrence (61). An important implication of a woman's response around the normalization of IPV is the effect it not only has on herself (staying in the relationship, acceptance of abuse, and barrier to seek help), but also on the community at large. If IPV is seen as normal, no one who is witnessing an incident would intervene (the so-called bystander nonintervention) (62). However, bystanders have the power to weaken perceived norms around the acceptability of IPV (63). Another important finding of the present study was the role children played in women's coping. Although some women expressed how their children helped them cope by being a source of inspiration and, thus, staying in the relationship, others recognized the adverse effects of the abuse on their children's health inspired them to leave the relationship. This points to the possibility that children of IPV survivors give them energy and strength to deal with the difficult situation they are enduring. These findings are consistent with previous literature highlighting the important role of children in the help-seeking behavior of IPV survivors by either contributing to staying in the relationship or leaving (64–66). Last, women in the present review reported motivating themselves as a means to cope with IPV. They mentioned what they had already accomplished so far and what was still out there for them to achieve. They, thus, exhibited resilience through their inherent motivations, which is consistent with literature suggesting that personal qualities are motivators helping individuals survive difficult situations (67, 68). Once again, this implies women's agency and strength in being able to motivate themselves even without external help to survive the pain they are going through.

### Limitations

Although this review has systematically evaluated the included studies and comprehensively examined the coping strategies used by women experiencing IPV in South Africa, limitations concerning the search strategies, number of studies, effectiveness of coping responses, and generalizability need to be considered. Further, limitations of the included studies need to be considered. The researcher of this review strived to conduct a systematic search strategy supporting the aims of the review as well as was possible. Even though an electronic search of databases is effective, it may only identify some of the eligible studies. To increase the likelihood of identifying more eligible studies, reference mining was added to the search method. Nonetheless, it cannot be ruled out that eligible studies were not identified and, thus, missed, which forms the first limitation. The second limitation is the small number of only 10 included studies, which concludes that the findings cannot be overgeneralized. This limitation also goes for the included studies as they were all conducted only in South Africa and only with women survivors. Further, this systematic review did not exclusively focus on the effectiveness of each of the coping responses in terms of well-being and helpfulness, which forms another limitation. Although the present review has cited literature that evaluates the mentioned coping responses in terms of their helpfulness, a closer look at coping strategies' effectiveness and their comparison is needed. This should be integrated into future research to see, which coping strategies, in fact, have the potential to mitigate the impact of IPV. Last, readers should note that this study focused solely on experiences of cis-gender women whose relationships were characterized as heterosexual. Thus, experiences of male or LGBTQ survivors of IPV were not included, making generalizability of results not possible, at least for males, non-cis-gender women, and non-heterosexual relationships.

### Recommendations

Based on the findings of the present study, the following three recommendations are made. First, agency at a collective level (community and society) needs to be facilitated. Our study has shown that IPV is a complex, multifaceted phenomenon, which occurs within a social context influenced by many levels. Although it is important for women experiencing IPV to be empowered to facilitate agency at an individual level, the multilevel influence on a woman's experience of IPV demand facilitation at the collective level as well. Facilitators should, for example, address structural barriers encountered by women, which hinder effective responses. The following examples may help in breaking those barriers: (1) Having regular visits and events from different organizations aimed at supporting IPV survivors in different places (to draw attention to an event and the organizations, leaflets could be spread), (2) a police workshop at which experts in the field of IPV and its consequences sit down with different police departments to discuss the issue and how women can more easily reach and talk to the police. Such workshops could be made mandatory. (3) Doctors use a fitted-out van to provide free medical checkups and care in different cities and villages. And, by that, survivors of IPV may be identified and referred for further assistance. Second, beliefs and norms of the society need to be challenged. The findings of the present study have shown that many women had internalized harmful gender roles putting them in subservient positions. They emphasized their duties as a wife and mother as well as their responsibility to keep the family together. Also, they normalized and minimized their experience with abuse. It, thus, seems imperative to start shifting the power balance within the relationship as well as the community, which contributes to those beliefs. The following are some ideas on how to do that: (1) advertisement, for example, portraying strong, self-confident and admirable single mothers in TV, billboards etc. to challenge the current view of a women being dependent on a man; (2) financial support for IPV survivors to also challenge the view of a women being dependent on financial support from a man; (3) fines/punishments for abusers, which are higher than the current ones to target the ineffectual handling of abusers in society today; (4) billboards highlighting the terrible facts about IPV. This may lead to more frequent public talks about the issue and eventually lead to a society that more openly addresses this issue. Last, women's emotions need be integrated in IPV services. Organizations providing services to survivors of IPV should recognize the influence of women's emotions in their decision making. Findings of the present study show that, for example, many women still love the man who is abusing them and feel attached to the life they have together. These factors need to be considered to properly assist women and help them to find their full independence.

### Future Research

Based on the findings of this systematic review, future research should address the following. First is the effectiveness of coping responses in terms of well-being. As mentioned above, one limitation of the present review was the in-detail analysis of the effectiveness of each reported coping strategy. However, this may be valuable information as it could inform organizations aimed at supporting survivors of IPV about the coping strategies that are actually helpful and effective. Second, the effectiveness of coping responses in terms of leaving the abuser. Further research may be used to not only examine how particular coping responses enable survivors to cope but also how they facilitate the process of leaving an abusive partner. Last, male and LGBTQ survivors' perspectives of coping experiences should be investigated as their lives and experiences are relevant as well.

## Conclusions

This qualitative systematic review was undertaken to better understand the coping experiences of women survivors of IPV. Ten studies were included in this review after a rigorous search and inclusion process. All included studies were of high quality based on the JBI Critical Appraisal Checklist for Qualitative Research (Table 1).

This review identified and provided an understanding of the different kinds of coping mechanisms used by South African women who experience IPV and compared them to available literature. Coping strategies in this study involved help- and support-seeking from others, emotional regulation efforts, and avoidance and distraction. Exploring these coping strategies pointed out the need for understanding IPV and responses to it within a broader context, which involves interpersonal, relationship, and communal as well as societal aspects. Hence, IPV constitutes a complex phenomenon, which is influenced by multiple factors. Although some coping responses are considered by previous literature as effective, others are not. The findings in this review confirm this as each theme had both effective and ineffective strategies. Nevertheless, the authors agree with previous research stating that seeking help and support from others (first theme) is an effective strategy. This also supports the abovementioned notion that IPV should be understood within an ecological system with the different levels involved in not only the emergence and perpetuation of IPV, but also in the resolution. Approaching IPV accordingly can lead to a change in societal norms, better access to and delivery of services to IPV survivors, and more functional family affairs as well as personal well-being and higher quality of life.

## Data Availability Statement

The original contributions presented in the study are included in the article/Supplementary Material, further inquiries can be directed to the corresponding author/s.

## Author Contributions

YS, NR, and RR contributed to the conception, design of the review, writing of the paper, and drafting the manuscript. YS and NR contributed to the literature search and analysis as well as the result interpretation. All authors contributed to the article and approved the submitted version.

## Conflict of Interest

The authors declare that the research was conducted in the absence of any commercial or financial relationships that could be construed as a potential conflict of interest.
