# Supplementary material for: Coping With the Experiences of Intimate Partner Violence Among South African Women: Systematic Review and Meta-Synthesis
Source: Front Psychiatry. 2021 May 26;12:655130. doi: 10.3389/fpsyt.2021.655130 (PMC8187566; doi:10.3389/fpsyt.2021.655130)
Supplement: Supplementary file 1 [file Data_Sheet_1.docx]

Appendix 1: Databases and related search strings

| Database | Search string |
| --- | --- |
| WoS | (intimate partner violence OR domestic violence OR abusive relationship) AND (South Africa) AND (coping OR responses OR  coping strategy OR coping mechanism OR experience) |
| PubMed | (intimate partner violence OR domestic violence OR abusive  relationship) AND (South Africa) AND (experiences) |
| EBSCOhost | (intimate partner violence OR domestic violence OR partner  abuse) AND (South Africa) AND (response OR coping) |
| Google Scholar | intimate partner violence AND South Africa |
