# Supplementary material for: Coping With the Experiences of Intimate Partner Violence Among South African Women: Systematic Review and Meta-Synthesis
Source: Front Psychiatry. 2021 May 26;12:655130. doi: 10.3389/fpsyt.2021.655130 (PMC8187566; doi:10.3389/fpsyt.2021.655130)
Supplement: Supplementary file 2 [file Data_Sheet_2.docx]

Appendix 2: Study findings with illustrations and assigned credibility level

*Slabbert, 2010*

**Finding 1:** Children helped these women to cope. [U]

“It is just my children are. They are very important to me. I love them very much. They are the reason I am going on.” (p.182)

**Finding 2:** Their children was their goal in life and helped them go on. [C]

“I cope to be there for my child. I want to give her the best I can. She is everything to me.” (p.182)

**Finding 3:** Religion is one way of helping them cope with their difficult situations. [U]

“The Lord carries me through this difficulty. Last week he supplied abundantly for me.“ (p.184)

**Finding 4:** They had hope and identified that as a coping mechanism. [U]

“I believe and hope that everything will come right and that I’ll be happy. It is hope that lets me go on.“ (p.185)

**Finding 5:** Respondents viewed themselves as survivors rather than victims. [U]

“I am a survivor. I experienced very difficult times in my life, but I survived every time.“ (p.191).

**Finding 6:** Some abused women are able to identify their own courage, wisdom and resilience and are able to view themselves as capable human beings. [C]

“I will get to the top, even if the pressure is sometimes too much. I am a fighter and he won’t get me down.“ (p. 191).

**Finding 7:** Participants indicated that alcohol helped them to forget and to cope. [U]

“‘(..) and then I take a doppie. That’s how I cope.” (p.193)

“You take a “doppie” and dance just to try to get through life.... And to forget the pain.“ (p.193)

**Finding 8:** Participants found their family, friends, and neighbours to be very supportive. [U]

“My mother is my biggest support. If it was not for her, I would not cope. She is always there for me (...).“ (p.7)

“I have two friends. They help me a lot and talk to me...The aunty over there is also very nice (..)”. (p.7)

**Finding 9:** The Church was noted as providing support and a way of coping (pastor & members of church) [U]

“There is a church sister that always helps me” (p.10)

“The pastor prays for me often and told me several times that I must leave him” (p.9)

**Finding 10:** Use of some form of Professional [U]

“‘My psychologist tells me regularly what a strong woman I am (..)” (p.12)

*Maselesel, 2011*

**Finding 11:** To cope with the abuser she finds excuses for her abuse. [C]

“At least he loves me, he did not mean to hurt me.” (p.3)

**Finding 12:** She denies that her partner is responsible for his actions. [C]

“His girlfriend/relative is the one who is influencing him to abuse me.” (p.3)

**Finding 13:** The victim blames herself that she is responsible for the abuse. [C]

“My husband is experiencing serious pressure. I shouldn’t have talked to him it was too soon”. (p.3)

**Finding 14**: She deserves to be treated in that way. [C]

“My husband is experiencing serious pressure. I shouldn’t have talked to him it was too soon”. (p.3)

**Finding 15**: She withdraws from her abuser. [C]

“I did not ask for his money because I did not want to be beaten.” (p.4)

**Finding 16:** Search for her identity. [C]

**“**How do I face the future(...) What will I do without him.” (p.4)

**Finding 17:** Victim accept there is nothing she can do about the abuse. [C]

“I have made a commitment to this marriage (..) I will not go away, he will have to kill me if he wants.“ (p.4)

**Finding 18**: Enduring the relationship [C]

“I have made a commitment to this marriage (..) I will not go away, he will have to kill me if he wants.“ (p.4)

**Finding 19:** Women retaliates and is prepared to fight. [U]

“I wish I could kill him before he kills me“ (p.5)

**Finding 20:** Search for meaning and a new understanding of their authentic self. [C]

“My kids need me, so I need to save myself from this animal. It’s not my fault that I am abused.” (p.5)

*Van der Merwe & Swartz, 2015*

**Finding 21:** Coping mechanism included religion. [U]

“God walks with you (...). I’m strong with him and that pulled me through.

**Finding 22:** Coping mechanism included support from family. [US]

/

**Finding 23**: Coping mechanism included counselling. [U]

“The counselling has made me stronger, really, really, because I kept a lot of things in me.” (p.8)

**Finding 24:** Coping mechanism included substance abuse. [US]

/

*Rasool, 2013*

**Finding 25:** Love women feel for the abuser [U]

“It was the feelings I still had for him for the duration of our marriage, 10 years and the abuse has been for 10 years (..)I truly felt that I love this man and I must stand by him“ (p.5)

**Finding 26:** abuser display caring and loving behavior. [U]

“‘First, he was angry and then [the] next second he held me, and he’s loving and tells me he loves me (...) he was my reason for living. I didn’t know what I’ll do without him, even though he was treating me like shit.” (p.6)

**Finding 27:** Hope that relationship will return to a better time. [C]

”I was too infatuated with the whole idea of our marriage, our partnership....I had high hopes [for our life].” (p.5)

*Mkhonto, 2014*

**Finding 28:** Resilient due to their inherent motivation. [C]

‘“(..) I am special (..) I have managed to reach grade 12, there are many chances that I can get so that I become better and become like other people.“ (p.12)

**Finding 29**: Resilient due to their spirituality [U]

“Sometimes I kneel and pray, and my emotions normalize.“ (p.12)

**Finding 30**: Children as a source of inspiration [C]

“(..) I wish (God) could add more days to my life so that I live for their sake (her children)’
(p.12)“

*Boonzaier, 2014*

**Finding 31:** Fighting back. [U]

“And after a while you just start thinking to yourself: “No this cannot go on.” And then you start fighting back.” (p.18)

**Finding 32:** Emphasized femininity or good womanhood (role as good wives, mothers and homemakers). [U]

“He needed me to clean him up, he needed me to take care of him, he needed me to take care of the mess he was in. He needed me to take care of his children and to fight his battles.” (p.20)

*Baholo, 2015*

**Finding 33:** They turned to family and friends during those times when they were desperate for support. [U]

“I got some support from my sister and my cousin(...) My sister also likes to defend me(..)”
(p.8)

**Finding 34:** effect of abuse on children. [U]

“He thinks you are the worst stupid ever and I think whoever is in the same relationship, they must quit for the sake of the children because it does not harm us only, but it affects the children: their schoolwork, their emotions, spiritually. They really get hurt. If I tell you about my kids, they really got scars in their heart.” (p.7)

**Finding 35**: anger at the abuse [U]

“I thought that I’m chasing nothing here; I was very angry at him (...)” (p.7)

*Rasool, 2015*

**Finding 36:** Fulfilling the role of mother [C]

“You see before I do something, I think about them. Who is going to show them what is right and wrong?” (p.10)

**Finding 37:** preserving the two-parent family form [C]

“I didn’t want my children growing up without a father“ (p.11)

*Dekel & Andipatin, 2016*

**Finding 38:** Internalization of dominant prescriptions of femininity [C]

“(..)I had to pamper him. I had to see that he is happy“ (p.11)

**Finding 39:** Participants believed that their partners could change [U]

“Well..I thought I could change him.” (p.12)

**Finding 40:** Drawing on police services for help and assistance. [U]

“I ran to the police station and made a case against him (..)“ (p.13)

**Finding 41:** Positioning with religious discourses [U]

“I was like Gods wants me to be here(..)“ (p.15)

**Finding 42:** Maintain the romantic fairy-tale ideal (love) [C]

"He just took out this cutting knife and he just started cutting me and I didn't go to the doctor, he doctored it himself. He bathed me and literally pick me up and put me in the water and I said to myself 'he must really care for me, he loves me'" (p.18)

**Finding 43:** Many women dissociate the abuse from the “real man” and attributing it rather to factors that he does not have control over [C]

“He would grab me by my hair, he would drag me outside, and he would hit me, but he would be under the influence of drugs" (p.19)

**Finding 44:** IPV is normalized [U]

"I thought that, the abuse, that maybe it's part of life and I'd told myself, 'well, maybe it should be like that'" (p.22)

**Finding 45:** The women tended to blame themselves for their partner's violence, and accepted their partners' blaming of them [U]

"I was more blaming it [the abuse] on myself, well he says that, it's because of me that he's beating me and I was blaming myself, well maybe it's me, maybe I should listen to him more, you know things like that, so, yeah, I blamed myself actually"

*Chikwira, 2019*

**Finding 46:** Normalisation of IPV [U]

“(...) It’s like we as black people, black women, it’s like its normal to be abused by men...Its normal.” (p.60)

**Finding 47:** Minimization of IPV [U]

“The hitting is not so bad because the hitting I can hide, and I can lie about it(..)You get used to it. For you its life...Permanent life.” (p.58)

**Finding 48:** Place blame of experiencing IPV on themselves (self-blame) [C]

“I always went for men that had the tendency of abusiveness or would fill up the void inside me that I thought I needed a man.” (p.52)

**Finding 49**: love influences a woman’s commitment to remain in a relationship, albeit an abusive one [U]

“I just kept it in for, for, for so long, like. I didn’t give up when it started, I still wanted to continue with the relationship because, I loved him and...I really didn’t know it was abuse at that time.” (p.54)
