# Supplementary material for: Coping With the Experiences of Intimate Partner Violence Among South African Women: Systematic Review and Meta-Synthesis
Source: Front Psychiatry. 2021 May 26;12:655130. doi: 10.3389/fpsyt.2021.655130 (PMC8187566; doi:10.3389/fpsyt.2021.655130)
Supplement: Supplementary file 4 [file Data_Sheet_4.docx]

Appendix 4: Participants Characteristics

| Study | Nr. of women | Ethnicity | Age | Children  Yes/no | Employment |
| --- | --- | --- | --- | --- | --- |
| Slabbert, 2010 | 20 | Black= 5 Coloured= 12 White = 3 | 23 - 49 | Yes= 20 No= / | n/a |
| Maselesel, 2011 | 18 | Black= 18 | n/a | n/a | Employed= 4 Unemployed= 6 Other= 8 |
| Van der Merwe & Swartz, 2015 | 4 | Coloured= 4 | 22-54 | n/a | n/a |
| Rasool, 2013 | 17 | n/a | n/a | n/a | n/a |
| Mkhonto et al., 2014 | 10 | All Tswana | 21-50 | Yes= 8 No= 2 | Employed= 4 Unemployed= 6 |
| Boonzaier, 2014 | 44 | Black= 43  Coloured= 1 | n/a | n/a | n/a |
| Baholo et al., 2015 | 11 | Black= 10  Indian= 1 | n/a | n/a | Employed= 5 Unemployed= 6 |
| Rasool, 2015 | 17 | Black= 6 Coloured= 9 White= 1 Indian= 1 | 19- 46 | n/a | Employed= 8 Unemployed= 9 |
| Dekel & Andipatin, 2016 | 7 | Black= 6 Coloured= 1 | Mean = 38 | Yes= 7 No= / | Employed= 2 Unemployed= 5 |
| Chikwira, 2019 | 11 | Black= 2 Coloured= 8 White= 1 | 21-41 | n/a | Employed= 3 Unemployed= 8 |
